# Vocational rehabilitation and return to work after spinal cord injury: a scoping review of policies in the U.S. and Canada

**DOI:** 10.3389/fresc.2026.1814254

**Published:** 2026-06-08

**Authors:** Georgios Theotokatos, Patrick Reardon, Jiaqi Chen, Kang An, Yuxiang Liu, Reuben Escorpizo

**Affiliations:** 1 Department of Physiotherapy, School of Health and Care Sciences, University of West Attica, Athens, Greece; 2 Department of Rehabilitation and Movement Science, The University of Vermont, Burlington, VT, United States

**Keywords:** disability rights, employment policy, human functioning, return to work, social inclusion, spinal cord injury, supported employment, vocational rehabilitation

## Abstract

**Purpose:**

Spinal cord injury (SCI) significantly impacts quality of life and economic independence, yet employment rates remain disproportionately low, suggesting a divergence between the legislative intent of inclusion and the clinical goal of functioning. The alignment of social policy with rehabilitation practice is essential. This study conducted a comparative scoping review of federal policies and policy-driven vocational rehabilitation (VR) strategies in the U.S. and Canada to map the evidence landscape and identify critical gaps in cross-national research.

**Methods:**

The PRISMA guidelines and the Arksey and O'Malley framework were used. We searched Ovid Medline, CINAHL, and Google Scholar, using COVIDENCE for the final review. Guided by the Population-Concept-Context (PCC) framework, we included English-language studies and materials that addressed federal-level policies in Canada and the U.S. related to the employment of persons with SCI.

**Results:**

Overall, 10 studies were analyzed. The literature predominantly focused on the U.S., particularly within the Veterans Health Administration (VHA) and state VR systems. A recurring barrier identified across both nations was the “benefits trap,” where social protection policies create financial disincentives that affect return-to-work (RTW). On the other hand, integrated interventions such as Individual Placement and Support (IPS) and federally mandated resource allocation (e.g., for assistive technology) demonstrated superior outcomes compared to traditional models.

**Conclusions:**

Current policy frameworks, while effective at protecting against discrimination, are often insufficient to overcome structural and financial disincentives to employment. To truly reconcile the goals of rehabilitation and inclusion, future efforts must address policy-driven barriers and prioritize funding for integrated, hospital-based vocational services that translate legal rights into employment outcomes for individuals with SCI.

## 1 Introduction

Spinal cord injury (SCI) affects the physical, social, and economic aspects of life. In the United States (U.S.), studies have shown 54 cases per million and the prevalence rate was 721 to 906 per million people (1). Cause of SCI can be traumatic (e.g., road injuries, sport-related injuries, terrorism or violence) or non-traumatic (e.g., infections or vascular damage) resulting in the interruption of neural pathways between the brain and body (2). The consequences of SCI vary depending on the level and severity of injury, but the impact on daily functioning remains and severe disability may ensue. Common SCI-related impairments include decreased sensation and motor control, bowel and bladder management limitations, spasticity, pain, or sexual dysfunction. Beyond the physical consequences, individuals with SCI often experience mental health challenges, including anxiety, depression, post-traumatic stress disorder, and substance use disorders (3). Therefore, SCI has a multifactorial effect on functioning, participation in the community and quality of life. Functioning is a complex concept encompassing major aspects of life, including the ability to move around, to work, access to education and transportation, to care and services, fulfilling family roles, and community integration. In practice, there is often a conflict between the goals of clinical rehabilitation and the reality of social policy: More specifically, when social protection policies inadvertently create disincentives for economic participation (4, 5, 34).

People with SCI also face significant barriers to community reintegration and employment. Gaining or retaining employment after SCI is particularly challenging. SCI-related disability, limited activities, and restricted participation can significantly affect one's ability to return to or remain in the workforce (6). In a recent 22-country international survey revealed an average worldwide SCI employment rate of only 38%, highlighting gaps compared to general population employment rates (7). Furthermore, these vocational challenges are not experienced equally across the SCI population. Historically, racial minorities, women, and individuals relying on disability benefits face structural barriers that disproportionately hinder their RTW. For example, African Americans with SCI experience significantly lower odds of employment and substantial earning disparities compared to their White counterparts (8, 9). As Post et al. (7) suggested, these broad employment disparities underscore how systemic and country-level factors, such as inconsistent healthcare access, lack of tailored vocational resources, and complex benefit systems, actively penalize vulnerable subgroups. In turn, this affects their productivity and capacity to earn because of either lack or reduced employment. Employment plays a critical role in self-determination, mental health, and perceived well-being. For individuals with SCI, work provides not only financial independence but also social connection and purpose (10, 11). Addressing employment inequities requires systemic change, enhancing workplace accessibility, improving inclusive policies, and investing in educational and vocational training initiatives that support sustained employment among individuals with disabilities (12).

Many individuals rely on federal disability benefits, with an estimated 63%–67% of people with SCI receiving support within six years of injury (12). Compared to the general population, people with SCI are 28% less likely to receive pay for work and 36% more likely to depend on disability benefits. These figures reveal persistent disparities in work participation, while the patients face long-term high medical costs associated with SCI, averaging $21,450 per patient for acute treatment, and reaching up to $132,807 for those with high tetraplegia (13). Long-term expenses are even greater due to frequent readmissions for complications, such as urinary tract infections, respiratory issues, pressure ulcers, and autonomic dysreflexia further compounding the life-long impact of SCI.

Despite significant advances in medical care, rehabilitation technologies, and policy initiatives, employment rates among people with SCI remain disproportionately low compared to the general population (14). The RTW process is influenced by a complex interplay of medical, psychosocial, and systemic factors. Barriers such as physical or cognitive impairments are directly injury-related, while others stem from environmental hindrances, such as workplace accessibility, employer and co-worker attitudes, and the structure of VR services. Interventions (some of which may be driven by policies or law) including VR, supported employment models like Individual IPS, assistive technologies, and benefits counseling have shown varying levels of effectiveness in improving RTW outcomes.

Recent analyses of SCI data across 22 countries (10) revealed significant between-country variability, driven by differences in Gross Domestic Product, infrastructure, and policy frameworks, in determining both the quality of life and perceived health of individuals with SCI. Importantly, perceptions of well-being or health and quality of life were strongly associated with employment status. However, the extent to which national and regional policies influence these outcomes remains unclear, highlighting the need for a comparative study on policies.

### 1.1 Policy overview: U.S. and Canada

In the U.S., the Americans with Disabilities Act (ADA) of 1990 marked a landmark advancement in protecting the rights of individuals with disabilities. The ADA suggests employers with 15 or more employees to provide equal opportunities in hiring, advancement, and benefits (15). Earlier groundwork was laid by the 1920 Smith-Fess Act (16), which extended VR programs, originally designed for disabled war veterans, to all individuals with disabilities (17). These programs promoted job training, skill development, and return-to-work support.

In Canada the Employment and Assistance for Persons with Disabilities Act (18) established employment rights, employer responsibilities, and job placement programs. The Act also created incentives for employers, such as wage subsidies and job training opportunities, to encourage the hiring of people with disabilities. More recently, the Accessible British Columbia Act (19) required public-sector organizations to meet accessibility standards and formed provincial accessibility committees to identify and remove barriers in employment, education, communication, transportation, health, and the environment.

In the U.S., approximately 305,000 individuals are living with an SCI. Data from the National Spinal Cord Injury Statistical Center (20) indicates that employment rates drop significantly post-injury; because this database tracks the overall traumatic SCI population regardless of medical fitness to work, it reports that only about 19% are employed at one year post-injury (20). Similarly, in Canada, approximately 40,000 individuals live with SCI, but only 38% of those aged 15–64 is employed compared to 70% of the general population (5). Canada introduced legal protections earlier than the U.S., beginning with the Canadian Human Rights Act of 1977 (21) and further strengthened by Section 15 of the Canadian Charter of Rights and Freedoms in 1985 (22), which guaranteed equal protection under the law for people with disabilities.

Given the impact of SCI on functioning, well-being, and participation, and recognizing employment-related disability, this scoping review aims to conduct a comparative analysis of relevant policies and policy-driven VR interventions between Canada and the U.S. (federal level). We explicitly included program evaluations and intervention studies (e.g., Supported Employment, IPS). By analyzing these interventions, we aimed to examine how overarching policy designs and accessibility frameworks are operationalized in practice, and how these VR systems ultimately affect RTW and stay-at-work outcomes among people with SCI in two high-income and well developed countries.

## 2 Materials and methods

### 2.1 Study design

A scoping review methodology was chosen to map the broad literature regarding legislation and vocational programs. We followed the principles outlined in the foundational framework by Arksey, O'Malley (23), and Johanna Briggs Institute (24). Reporting was aligned with the Preferred Reporting Items for Systematic Reviews and Meta-Analyses (PRISMA) guidelines for scoping reviews (25), where appropriate. The protocol for this scoping review was not pre-registered in the Open Science Framework (OSF) or any other public registry.

### 2.2 Eligibility criteria

Eligibility criteria were developed *a priori* using the JBI-recommended Population, Concept, and Context (PCC) framework. More specifically: 1) Population: individuals living with a SCI, 2) Concept: policies and policy-driven interventions related to the employment of persons with SCI, with employment as a reported outcome, 3) Context: policies applicable to the federal level in Canada and the U.S. Studies were excluded if they were opinion, perspective, or editorial, systematic review and/or meta-analysis. The selected countries are high-income nations that share strong, rights-based legislative frameworks protecting individuals with disabilities. However, they utilize fundamentally different healthcare delivery and social protection systems, allowing for a comparison of how different policy structures translate into employment outcomes. Additionally, the literature search included studies published from database inception up to March 2025.

### 2.3 Information sources and screening of studies

After consulting with a research librarian, we developed targeted and encompassing search strategies for three databases and included them here in the appendix (see Supplementary Material): OVID Medline (Supplementary Appendix A), CINAHL (Supplementary Appendix B), and Google Scholar (Supplementary Appendix C), all of which were saved into the COVIDENCE web-based software (26). We used hand searching of the references to the studies/materials found and the Web of Science to find any additional studies that cited the primary studies found in PubMed and CINAHL. While policy documents are frequently found in grey literature, our search was purposefully restricted to peer-reviewed academic databases. This decision aligns with our objective to evaluate policies specifically through the lens of scientifically evaluated vocational interventions, ensuring that all included literature had undergone rigorous methodological peer-review. Two independent researchers (with background in rehabilitation sciences) screened the title and abstract against the inclusion and exclusion criteria. Following the above procedure they also screened the full text of the studies or materials based on the PRISMA guidance for scoping reviews. To ensure reliability and consistency between reviewers the team engaged in preliminary calibration discussions to ensure mutual understanding of the extraction criteria. Any disagreements between the two independent reviewers during the title, abstract or full-text screening phase, were first discussed to reach a consensus. If an agreement could not be reached, a third independent reviewer was consulted to make the final inclusion or exclusion decision.

### 2.4 Data extraction and synthesis of findings

The information extracted focused on the policy title, author, year, effective period, policy objective, policy key points specific to RTW and Stay-at-work (SAT) for people with SCI in terms of processes, how policies directly impacted the lived experience of people with SCI (as reported in the included qualitative studies) eligibility, benefits, and specific outcomes targeted by the policy (executed March 2025). Findings were summarized and synthesized according to overlying themes related to the policies’ applicability (population), employment barriers and facilitators addressed by the policies, and benefits to the employment of people with SCI.

## 3 Results

### 3.1 Retrieved studies

**Figure 1:**
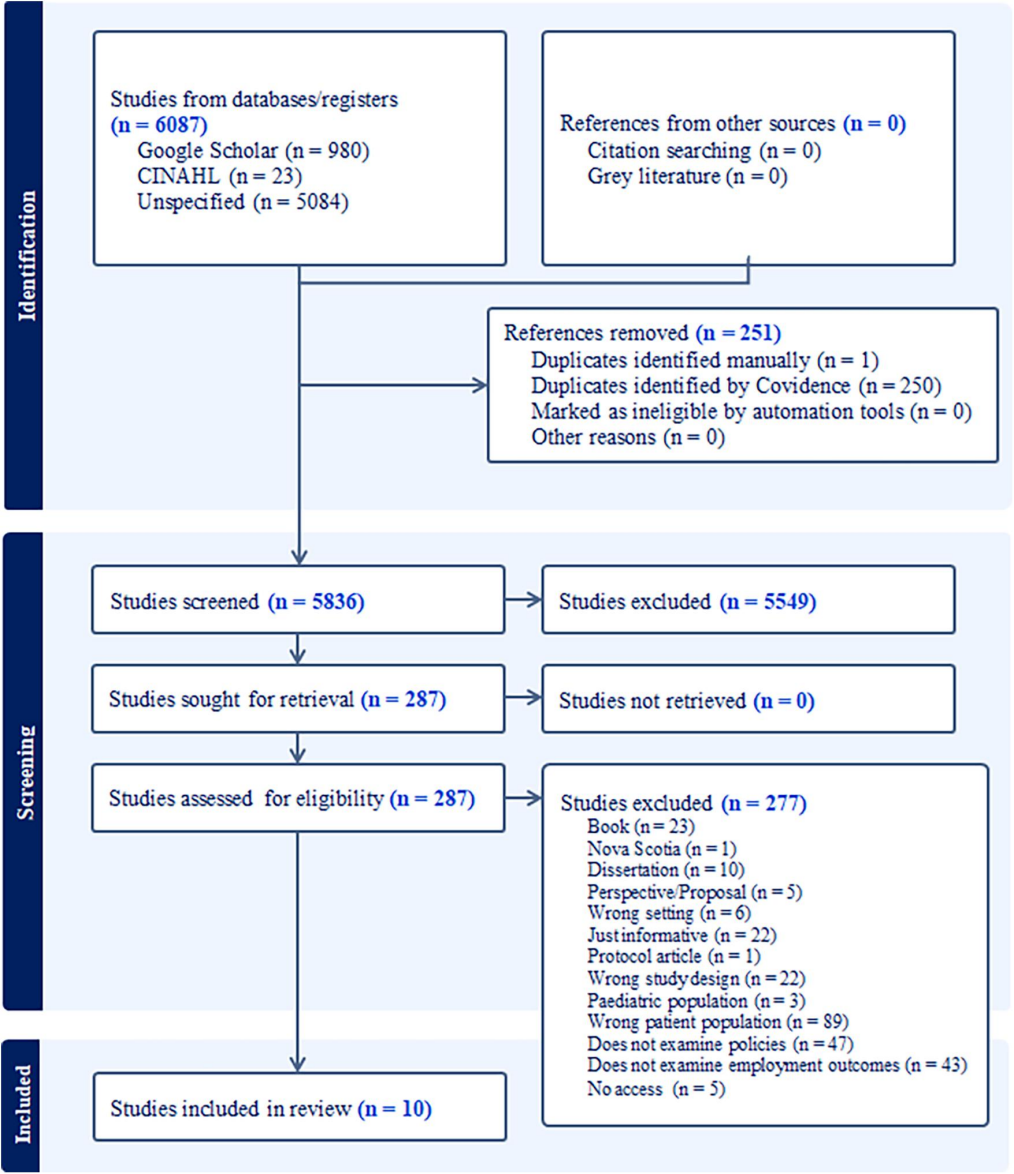
PRISMA flow diagram illustrating the selection process for the scoping review.

A total of 6,087 studies were initially imported for screening through COVIDENCE. Upon removal of duplicates, there were 5,836 studies left for screening, of which 10 were included after full-text screening and consensus (final reviewer agreement = 100%) based on the inclusion and exclusion criteria (PRISMA flowchart can be seen in Figure 1). The studies range from early investigations of benefit disincentives (4) to modern evaluations of evidence-based supported employment (14).

### 3.2 Population characteristics

The primary aim of these studies was to evaluate the effectiveness of laws, programs, and VR interventions in improving employment outcomes for individuals with SCI. The included studies used diverse methodologies (randomized controlled trials, longitudinal observational studies, qualitative interviews, retrospective analyses) and sample sizes, based on their focus and aims. The participants of the studies were predominantly males, reflecting the demographics of the SCI. For example in the Veterans Health Administration studies, approximately 97% of the participants were males (27, 28), while the Rehabilitation Services Administration database study reported approximately 69% males (29). In Wilbanks & Ivanova work (30), three out of four participants had cervical injuries (tetraplegia), while in Ottomanelli et al. (28) work nearly 40% of participants had motor complete injuries, and 59.2% had a history of traumatic brain injury.

The summary of the retrieved studies and population characteristics can be seen in Table 1.

**Table 1:** Characteristics of the retrieved studies.

Author (Year)	Country	Design	Sample size	Primary focus
Better et al. (4)	U.S.	Retrospective analysis	*N* = 65,155	Impact of disability benefits on rehabilitation outcomes
Inge et al. (31)	U.S.	Case study	*N* = 3	Assistive technology and workplace supports for tetraplegia
Atwell & Hudson (34)	U.S.	Policy/Descriptive	Policy description/case study	Social Security Legislation (Ticket to work)
Jongbloed et al. (5)	Canada	Qualitative (Survey)	*N* = 357	Impact of Government policies in Canada (British Columbia)
Cotner et al. (32)	U.S.	Mixed methods	*N* = 34	Facilitators and barriers in Evidence-Based Supported Employment (EBSE)
Inge et al. (29)	U.S.	Retrospective database	*N* = 9,205	RSA-911 employment outcomes (2011–2013)
Ottomanelli et al. (27)	U.S.	Secondary analysis of RCT	*N* = 81	Association of specific vocational activities with employment.
Wilbanks & Ivankova (30)	U.S.	Qualitative pilot	*N* = 4	Lived experience of RTW and Federal-State Vocational Rehabilitation Partnership (Rehabilitation Act)
Ottomanelli et al. (28)	U.S.	Longitudinal Observational	*N* = 213	Outcomes of 24-month Individual Placement and Support (IPS) program
Ottomanelli et al. (14)	U.S.	Retrospective analysis	*N* = 619,859	30-year perspective on ADA and employment trends using Recent Medical Expenditure Panel Survey (MEPS) data

### 3.3 Interventions

To clearly map the current evidence, the retrieved policies and interventions were categorized into three primary approaches based on their mechanism of support:

#### 3.3.1 Vocational rehabilitation (VR)

Traditional VR models were compared with integrated models with respect to outcomes and service delivery methods. Analysis of national U.S. data from 2011 to 2013 indicated a rehabilitation rate of approximately 50%–51% for individuals who had an Individualized Plan for Employment (IPE) implemented (29). However, retrospective data from 1979 highlighted a disparity in outcomes based on benefit status, indicating that beneficiaries of disability insurance were significantly less likely to be rehabilitated (55.6%) compared to non-beneficiaries (71.4%) (4). While this historical study highlights the early recognition of this disparity, more recent literature in our review confirms that this trend persists. Fear of losing benefits (5) and difficulties in navigating complex state vocational agencies continue to act as major variables to successful rehabilitation and employment today (14). Qualitative findings by Wilbanks and Ivankova (30) highlighted that state VR agencies were crucial for providing “early-training resources,” specifically by covering costs for vehicle modifications and durable medical equipment, which participants identified as essential for facilitating independence (30). In contrast, a secondary analysis of vocational activities found that office-based vocational counseling was less likely to be associated with employment success, compared to community-based activities such as job development (27).

#### 3.3.2 Supported employment (SE) and individual placement and support (IPS)

Studies focused on the IPS model, also referred to as Evidence-Based Supported Employment (EBSE), demonstrating its effectiveness over traditional methods. A longitudinal study of a 24-month IPS program integrated into SCI care reported a 43.2% competitive employment rate across all participants (Veteran population) (28). When analyzing a sub-sample of outpatients who did not have a history of traumatic brain injury (TBI), the employment rate exceeded 50% (52.2%). Success in supported employment was associated with specific provider activities. “Job development” (defined as networking with employers) and “job placement” were positively associated with employment outcomes, whereas general vocational counseling was more frequently associated with unemployment (27). Another crucial facilitator was the integration of the Vocational Rehabilitation Specialist (VRS) into the medical team (32). This way the medical providers were able to address rapidly health-related barriers that might affect employment.

#### 3.3.3 Assistive technology (AT) & benefits counseling

The included studies show the importance of the AT and the financial benefits as important factors in the RTW process. More specifically, Inge *et al*., had recognized the importance of AT as early as 1998. The AT is essential for independence for individuals with SCI. Successful work integration requires adaptations ranging from desk adjustments/modifications to devices with voice-activated software (31). The fear of losing benefits is an important barrier in both U.S. and Canada. In the USA beneficiaries of Social Security Disability Insurance (SSDI)/ Supplemental Security Income (SSI) were found to be rehabilitated less frequently than non-beneficiaries (4). In Canada, complex income support systems often deter return to work due to the risk of losing medical benefits like prescriptions and dental care once earnings exceed a threshold (5). This illustrates a systemic paradox where the safety net meant to ensure inclusion (via financial protection) inadvertently hinders the rehabilitation goal of functioning (work integration).

The policy-linked programs/interventions are shown in Table 2.

**Table 2:** Policy-linked programs and their impact on persons with SCI employment.

Policy/Program	Jurisdiction	Key features	Impact on persons with SCI employment
Americans with Disabilities Act (ADA)	U.S.	Prohibits discrimination. Promotes reasonable accommodations.	Participants injured pre-ADA noted it was “scary looking for a job” without these protections (30)
Ticket to Work and Work Incentives Improvement Act (WIIA)	U.S.	Allows beneficiaries to purchase VR services and extends Medicare coverage.	Designed to reduce the fear of losing health coverage, specifically expanding Medicare coverage for 93 months post-trial work period (34)
Employment Equity Act	Canada	Focuses on anti-discrimination and individual rights.	It addresses discrimination but fails to address structural barriers like poverty and lack of supports (5)
State VR Agency Funding	U.S.	Financial support for “big ticket” items such as vehicle modifications (including accessible vans) and home modifications, provided these items are determined to be necessary to achieve an individual's specific employment goal.	Identified as a primary facilitator for physical access to the workplace (30)

### 3.4 Outcome measures

Employment status outcome can be defined with various ways. Ottomanelli et al. (27, 28) defined employment outcome as jobs paying at least minimum wage in the competitive labor market. Better et al. (4) and Inge et al. (29) relied on the systems' standardized closure codes: Status 26 (Successful rehabilitation) vs. non rehabilitated closures (Status 08, 28, 30). Other approaches were the key economic outcomes which included weekly earnings and hours worked per week (29), and the reduction or retention of disability benefits (SSDI/SSI) (34). Finally, qualitative studies (5, 30) measured outcomes in terms of perceived barriers, facilitators, and the lived experience of returning to work, often highlighting the psychological impact of potential benefit loss.

## 4 Discussion

This scoping review synthesizes policies and VR interventions for persons with SCI in the U.S. and Canada. The findings highlight a divergence between the legislative intent of inclusion and the statistical reality of low employment rates, while identifying specific intervention models that are promising. Identifying successful and unsuccessful approaches enables improvements in employment access, equity, and retention.

Following data synthesis, variability was found in target populations, study designs, and outcome measurements. Despite the differences, consistent patterns emerged across studies. In the U.S., several risk factors for poor employment outcomes were identified, while certain VR programs demonstrated measurable improvements in RTW rates. In contrast, limited research exists regarding RTW and stay-at-work policies in Canada, with only one Canadian study meeting the set inclusion criteria, compared to 9 retrieved studies U.S. on the U.S., showing a major research gap. With only one Canadian study meeting the inclusion criteria compared to nine U.S.-focused studies, true cross-national comparative analysis was severely limited. However, this imbalance is a significant finding in itself, exposing a massive empirical research gap in how Canadian federal and provincial disability policies are evaluated in peer-reviewed literature.

Assistive technology, when tailored to individual needs, further expands feasible job roles, particularly in telecommuting and knowledge-based work (29, 31). The findings highlight the benefit of individualized, integrated interventions that address both physical and psychosocial readiness. While federal policies like the ADA establish a legal framework for inclusion, Wilbanks and Ivankova (30) demonstrate that legal rights alone do not guarantee functioning. Their findings indicate that RTW relies heavily on the federally-mandated, state-administered Vocational Rehabilitation system to fund “big ticket” enablers (e.g., Accessible vehicles) effectively bridging the gap between abstract rights and concrete resources.

While this scoping review maps several systemic barriers and facilitators, the policy implications drawn from this small and heterogeneous evidence base must be interpreted with caution, as our findings represent preliminary observations. Legislation such as the Americans with Disabilities Act (ADA) and the Ticket to Work and Work Incentives Improvement Act (TWWIIA) improved legal protections and reduced disincentives to employment. However, inconsistent enforcement, employer misconceptions, and complex benefit systems continue to limit their impact (14). Similarly, the Employment Equity Act in Canada mandates employer equity plans, but lacks accountability mechanisms, leading to limited real-world implementation (5). Both the U.S. and Canada could benefit from mutual policy learning, strengthening enforcement mechanisms and aligning benefit structures to provide incentives of sustained employment.

Several promising models and policy reforms were identified, such as Supported Employment and Individualized Employment Plans. Supported employment interventions have been shown to improve RTW outcomes (31), while individualized employment models emphasizing competitive, integrated work achieved higher income and satisfaction (28). These findings suggest the potential effectiveness of person-centered, technology-assisted, and policy-supported approaches to employment for people with disabilities. Veterans with SCI face unique psychological challenges, including higher rates of Post-traumatic stress disorder (PTSD), depression, and anxiety from combat exposure (28, 32). However, integrated care models within the Veterans Health Administration combining vocational, psychological, and peer support appear to improve overall engagement and employment outcomes.

In contrast, civilians often experience fragmented care, with separate providers for mental health and vocational services, reducing overall program efficacy. Beyond diagnosed conditions, factors such as self-efficacy, intrinsic motivation, and perceived control significantly influence RTW readiness. Civilians may struggle with self-doubt and job insecurity. Addressing these motivational dimensions appears critical for sustained RTW success. Findings from case and observational studies (30, 31) indicate that psychological readiness is likely as critical as physical capacity for successful RTW. Interventions combining VR with psychological counseling, peer support, and motivational enhancement demonstrate stronger and longer-lasting employment outcomes across populations. This highlights the need for integrated biopsychosocial models of rehabilitation that promote both employment and holistic well-being. This integration is essential for reconciling the goals of rehabilitation and inclusion. It moves the focus from a medical model of “fixing” the individual to a biopsychosocial model that aligns clinical recovery with social participation.

Future directions: Current literature identifies predictors of employment such as education, injury characteristics, the work's nature and complexity. Nevertheless, more analytical tools may be necessary to clinical decision and policy. In a recent scoping review authors suggested that Machine Learning (ML) offers a beneficial approach to identifying robust predictors of RTW by integrating large, heterogeneous datasets (33). Unlike traditional models, ML approaches can process complex interactions between personal, environmental, and medical factors, potentially enabling a “Learning Health System” that continuously improves the timing and targeting of vocational interventions. Integrating such data-driven approaches could help policymakers move beyond “one-size-fits-all” programs toward precision rehabilitation models that account for the complex health conditions.

Several limitations should be considered when interpreting the findings of this scoping review. First, the generalization of the results should be approached with caution. Second, regarding the literature search, we did not include grey literature. While grey literature is often utilized in policy-oriented reviews, we restricted our criteria strictly to peer-reviewed databases to ensure that the policy impacts and interventions discussed had undergone rigorous methodological evaluation. Finally, while the search strategy utilized in Google Scholar might appear brief, it was constrained by the platform's strict 256-character limit for search queries.

## 5 Conclusion

Successful RTW for individuals with SCI requires a coordinated approach combining medical rehabilitation, vocational training, psychological readiness, and enforceable policy support. Evidence-based models such as IPS, individualized employment planning, and assistive technology consistently improve employment outcomes, particularly when paired with long-term follow-up and tailored job matching. Both the U.S. and Canada have anti-discrimination laws and vocational rehabilitation frameworks, yet enforcement remains weak, leading to ongoing inequities. Racial minorities and women with SCI continue to face poorer RTW outcomes due to compounded systemic biases and a lack of tailored vocational resources. In addition, disability benefit recipients face the “benefits trap,” where the fear of losing essential healthcare coverage directly penalizes employment efforts. The lack of research in Canada underscores an urgent need for expanded study and policy evaluation. Future efforts must emphasize integrated, person-centered interventions that merge psychological and vocational supports, exploring ways to strengthen accountability for disability employment laws, and promote individualized employment pathways. Greater investment in research and enforcement across both regions will likely be essential to ensure equitable, sustainable employment opportunities for people with SCI and other disabilities.

## Data Availability

The original contributions presented in the study are included in the article/Supplementary Material, further inquiries can be directed to the corresponding author/s.
